# Reviewed article: Martins CI, Silva CSO, Souza FG, Faria SMC, Silva LR, Camargos MCS. Sociodemographic profile of elderly people cared for at a medium and high complexity hospital in Belo Horizonte, Minas Gerais, Brazil: a cross-sectional study. Epidemiol Serv Saude. 2025:34;e20240254.

**DOI:** 10.1590/S2237-96222025v34e20240254.a

**Published:** 2025-09-08

**Authors:** Jack Roberto Silva Fhon

**Affiliations:** Universidade de São Paulo

**Table:** 

Rating	Excellent	Good	Average	Below average	Poor
Interest				✓	
Quality				✓	
Originality				✓	
Overall				✓	

**Table:** 

Questionnaire	Yes	No	Not applicable
Does the manuscript contain new and significant information to justify publication?		✓	
Does the Abstract (Summary) clearly and accurately describe the content of the article?		✓	
Is the problem significant and concisely stated?		✓	
Are the methods described comprehensively?		✓	
Are the interpretations and conclusions justified by the results?		✓	
Is adequate reference made to other work in the field?		✓	

## Manuscript Structure

Length of article is: Adequate

Number of tables is: Adequate

Number of figures is: Adequate

Please state any conflict(s) of interest that you have in relation to the review of this paper (state “none” if this is not applicable): None

## Comments

Estudo importante mas que apresenta muitas limitações de estudo, em especial não responde o objetivo de estudo.

